# Quantifying the Frontal-to-Ceiling Domain Gap for YOLO-Based Hand Gesture Recognition in Smart Homes

**DOI:** 10.3390/s26185735

**Published:** 2026-09-09

**Authors:** Ufuk Beşenk, Sarp Ege Nayim, Ömür Öcal, Mehmet Öztemel, Ahmet Özkurt, Mustafa Alper Selver

**Affiliations:** 1 Department of Electrical and Electronics Engineering, Dokuz Eylül University, İzmir 35330, Türkiye; 2 Core Bina Otomasyon Teknolojileri San. ve Tic. Ltd. Şti., Fatih Mahallesi, Ege Caddesi, A2 Blok, No: 42/5, Gaziemir, İzmir 35410, Türkiye

**Keywords:** hand gesture recognition, frontal-to-ceiling domain shift, on-device inference, YOLOv8, smart home control, temporal verification, on-device privacy

## Abstract

Vision-based hand gesture recognition (HGR) systems are predominantly developed for frontal camera viewpoints, whereas smart-home cameras are often ceiling-mounted, creating a viewpoint-induced domain gap. To investigate this issue, we collected and manually annotated CeilGest, an 18-class ceiling-view hand gesture dataset comprising 156,282 annotated frames from 68 participants recorded in distinct domestic environments. We then systematically evaluated frontal-to-ceiling transfer using YOLO-based detectors trained on HaGRID and compared their performance with an in-domain CeilGest-trained model. On identical ceiling-view footage, the frontal-trained YOLOv8n produced approximately 24× more class-to-class misclassified frames than the in-domain model (486 vs. 20 across 27,000 frames); this large paired difference remained evident when temporal dependence within gesture holds was taken into account. The effect was strongly class-dependent, with AP decreasing by up to 5.5 percentage points for the worst-affected gesture, while the aggregate same-architecture mAP50 difference was 0.6 percentage points. Across five YOLOv8 variants evaluated on frontal HaGRID, mAP50 remained at 0.995, supporting selection of the lightweight YOLOv8n for edge deployment. The complete ceiling-view HGR pipeline was implemented on Raspberry Pi 5 using NCNN and Jetson Orin Nano using TensorRT. Mean inference latency was 74.69 ± 4.72 ms and 12.72 ± 0.12 ms, respectively. During a 10 min continuous Raspberry Pi 5 test, mean inference latency increased by 17.6% and junction temperature reached 90.8 °C with thermal throttling. Power consumption and INT8 inference were not evaluated. Overall, the results show that training–deployment viewpoint consistency is a major consideration for ceiling-mounted HGR and establish an in-domain supervised baseline relative to frontal-only training without domain adaptation.

## 1. Introduction

Hand Gesture Recognition (HGR) is a compelling modality for smart-home control with contactless, low cognitive load. It is potentially relevant for individuals with speech or mobility impairments for whom voice assistants or touchscreens present barriers [1,2,3,4,5]. Single-stage YOLO detectors achieve up to 0.99 mAP_50_ on HGR benchmarks such as the 18-class HaGRID [6,7,8] and the TSL Detection dataset [9].

However, nearly all vision-based HGR systems assume frontal camera perspective [10], yet smart-home cameras are routinely ceiling-mounted for wider coverage. This mismatch creates a measurable domain gap. On identical ceiling footage, a frontal-trained detector produced ≈24× more misclassified frames than one trained on in-domain data (486 vs. 20 misclassified frames across the 27,000-frame test set; see Section 4). On the worst-affected class (i.e., call), per-class mAP_50_ fell by up to 5.5 percentage points.

Furthermore, deploying these models in real smart homes introduces four interconnected challenges: (i) selecting an expressive yet intuitive and user-friendly gesture vocabulary; (ii) overcoming the viewpoint-induced domain gap between frontal training data and ceiling-mounted cameras; (iii) achieving efficient inference on resource-constrained edge hardware; and (iv) preserving user privacy through fully on-device processing.

- Gesture vocabulary: We target 18 static postures mapped to home-automation commands. Static gestures avoid temporal-modelling overhead on edge hardware, align with the HaGRID class definitions [6], and impose lower motor demands on users with limited dexterity.
- Domain gap: Ceiling mounting introduces foreshortening, geometric distortion, and altered occlusion patterns [11], yet the overwhelming majority of HGR datasets assume frontal or first-person (i.e., ego-perspective) views [10].
- Edge deployment: Running models within 4–8 GB memory and 7–15 W TDP requires quantisation [12,13] and hardware-specific backends, which can degrade accuracy [14]. Despite this, only a small fraction of edge HGR studies target sub-15 W devices [15].
- Privacy: The GDPR [16] recognises that hand geometry may constitute biometric data when processed for the purpose of uniquely identifying a natural person; users therefore prefer local processing. This motivates a fully on-device pipeline where frames are processed and immediately discarded.

Within the literature surveyed in Section 2, we did not identify a system that jointly addresses these four challenges. Baptista et al. [17] show that user-identity shift degrades HGR accuracy from near-perfect to ≈84% and recover it to ≈90% via contrastive training, but only for four classes under a fixed frontal camera on a desktop GPU. Alabdullah et al. [4] achieve 92.6% accuracy on seven HaGRID classes using skeleton-feature fusion with a recurrent neural network (RNN), yet require desktop hardware and a frontal viewpoint. Hobbs and Ali [18]—the closest edge-deployed system—run MediaPipe landmarks through a TensorFlow Lite classifier on Raspberry Pi 5 at 20.4 FPS, but rely on CPU-only inference, a frontal camera, and a simple 3-of-4 majority vote for temporal filtering. However, their skeleton-based pipeline degrades under non-frontal angles [10]. GECO [19] demonstrates end-to-end IoT integration with sub-50 ms latency, yet is limited to frontal views on a smartphone processor.

Each of the above-mentioned studies addresses at most two of the four challenges; thus the intersection of frontal-to-ceiling domain shift, accelerated edge inference, and an 18-class vocabulary remains an open area of research.

Accordingly, the contributions of this study are twofold:

(1) Frontal-to-ceiling domain-gap quantification: We present what is, to the best of our knowledge, the first systematic analysis of the viewpoint-induced performance degradation of YOLO-based HGR under ceiling-mounted cameras. To support this analysis, we collected and manually annotated CeilGest, a ceiling-view hand gesture dataset comprising 156,282 images from 68 subjects, and evaluated the impact of viewpoint shift using both HaGRID and CeilGest.

(2) Privacy-preserving edge deployment: We develop and validate a complete smart-home HGR pipeline for on-device inference using YOLOv8 on Raspberry Pi 5 (NCNN) and Jetson Orin Nano (TensorRT), incorporating temporal verification to improve robustness while preserving user privacy through local processing.

The remainder of this paper is organised as follows. Section 2 reviews related work on HGR, viewpoint robustness, and edge AI. Section 3 describes the proposed system architecture, the HaGRID and CeilGest datasets, the training procedure, and the deployment pipeline. Section 4 presents the experimental results, including model-capacity analysis, frontal-to-ceiling domain-gap evaluation, and edge deployment validation. Section 5 discusses the findings, limitations, and future research directions. Finally, Section 6 concludes the paper.

## 2. Related Works

This section reviews HGR for smart-home control, viewpoint robustness and domain shift, and edge-AI deployment on resource-constrained hardware.

### 2.1. Hand Gesture Recognition (HGR)

HGR has progressed from handcrafted-feature methods [1,2] through convolutional neural network (CNN)–long short-term memory (LSTM) hybrids [20,21] to lightweight single-stage detectors and transformers [22], yet robust cross-environment generalisation remains an open challenge. Detection-based formulations in particular have been applied across both dynamic and static gesture settings, from SSD-style detectors for human–computer interfaces [23] to two-stage variants combining Mask R-CNN with multibox detection in applied domains [24]. Recent surveys converge on the limitations relevant here: Linardakis et al. [10] report that the overwhelming majority of studies assume a frontal camera and that bounding-box capture dominates classification pipelines; Cui et al. [3] find that lightweight models exhibit weak environmental adaptability; and Fertl et al. [15] find that only a small fraction of systems target genuine edge hardware. These observations—frontal bias, limited environmental robustness, and desktop dependence—define the gap addressed by this work.

#### 2.1.1. YOLO-Based Gesture Detection

Gao et al. [9] benchmarked YOLOv8n–v13n on 31 gesture classes, with YOLOv9t achieving mAP_50_ = 0.990 and YOLOv10n the fastest inference (0.7 ms), though on a single custom dataset. We selected YOLOv8n and YOLOv10n for their TensorRT compatibility, competitive nano-scale accuracy, and, for YOLOv10n, NMS-free inference that reduces edge latency [8].

#### 2.1.2. Smart-Home HGR Systems

Table 1 compares closely related methods. Alabdullah et al. [4] and Ahuja et al. [25] reach 92.6% and 93.0% classification accuracy but are limited to desktop-GPU, frontal-view settings with small vocabularies (7 and 10 classes). Hobbs and Ali [18], the closest prior edge-deployed system, use MediaPipe landmarks on a Raspberry Pi 5 (CPU-only) for 15 classes at ∼90% accuracy. Lopes et al. [19] present GECO, a MediaPipe-based gesture controller running on Android with MQTT-based IoT integration, achieving high user-rated intuitiveness (9.5/10) and <50 ms latency, but supporting only seven classes under frontal views without dedicated edge accelerators. A consistent pattern emerges: existing systems either achieve high accuracy on desktop hardware under frontal views, or deploy on edge with reduced accuracy and vocabulary. None evaluate the frontal-to-ceiling domain shift.

### 2.2. Viewpoint Robustness and Domain Shift

Camera viewpoint changes introduce geometric distortion, foreshortening, and altered occlusion that degrade models trained on a single view. The closest precedent is overhead person detection: Ahmad et al. [26] train a YOLO detector on frontal-view data and evaluate it on an overhead-view person dataset, confirming that the canonical-to-overhead shift is severe enough to warrant separate treatment. Their target is nonetheless the whole human body, whose overhead appearance is considerably more stable than that of a deformable hand pose. In the broader action-recognition literature, cross-view methods learn view-invariant representations to cope with drastic appearance changes between viewpoints [11]; however, these approaches target full-body actions captured by multi-camera rigs and do not transfer directly to single-camera HGR settings.

The 3D hand pose community has addressed viewpoint variation more directly—InterHand2.6M [27] and FreiHAND [28] show that hand appearance changes substantially across views due to self-occlusion and finger foreshortening—but these methods require depth sensors or multi-view setups [29] incompatible with single-camera edge deployment, and recover joint coordinates rather than gesture classes. For HGR specifically, Andriyanov and Mikhailova [30] evaluated MediaPipe + YOLO-Pose under moderate angle variations (mAP > 0.80) without considering ceiling perspectives.

Data augmentation including learned policies [31] can simulate moderate viewpoint changes but cannot fully recover the ceiling transformation. Domain-adaptation methods such as adversarial alignment [32] and knowledge distillation [33] offer more principled alternatives, yet to the best of our knowledge, no prior study has quantified this specific domain gap for hand gestures. In our experiments, collecting in-domain ceiling data and retraining proved highly effective (mAP_50_ > 0.99; Section 4); we deliberately scope this study to establishing the in-domain performance baseline rather than benchmarking DA strategies (Section 5.4).

### 2.3. Edge AI for Smart Environments

Edge computing reduces latency and enhances privacy versus cloud designs [34,35], but deploying deep models on constrained hardware requires pruning [12], INT8/FP16 quantisation [13], and knowledge distillation [33]; lightweight architectures have likewise been pursued for other dense prediction tasks such as semantic segmentation [36]. These are complemented by platform-specific engines: TensorRT for Jetson, NCNN [37] for ARM CPUs. YOLOv8 [7] and YOLOv10 [8] suit edge deployment owing to anchor-free heads, efficient backbones, and NMS-free inference. Alqahtani et al. [14] found the Jetson Orin Nano fastest and most energy-efficient among RPi 3/4/5 and Coral TPU configurations, though their benchmark covered general object detection (COCO) rather than HGR; among the reviewed HGR systems, Hobbs and Ali [18] report a 15-class skeleton-based recogniser on a CPU-only Raspberry Pi 5 at ∼90% accuracy.

Of reviewed HGR systems, only Hobbs and Ali [18] deploy on genuine edge hardware, and Lopes et al. [19] on mobile processors rather than dedicated accelerators. Accelerated inference backends are thus mature for general object detection, but their application to HGR, particularly under non-frontal viewpoints, remains unexplored.

## 3. Materials and Methods

### 3.1. Datasets

#### 3.1.1. Benchmark Dataset Selection and Prior Work

HaGRID [6] was selected as the source-domain dataset because it is currently the largest publicly available RGB hand gesture detection benchmark with bounding-box annotations. It contains 554,800 images collected from 37,583 contributors, covering 18 gesture classes together with a no gesture class. Its gesture vocabulary closely matches the requirements of smart-home control while its scale enables robust detector training.

A key limitation of HaGRID is that it was collected almost exclusively from frontal webcam viewpoints, which limits its suitability for evaluating ceiling-mounted cameras (see Section 4). This frontal-view bias motivates the collection of the CeilGest dataset described in the following subsection.

Alternative datasets such as Jester [38], NVGesture [39] (dynamic gestures, no bounding boxes), and EgoGesture [40] (first-person views) were not selected because they target different recognition tasks, including dynamic and egocentric gestures, and therefore do not provide an appropriate benchmark for ceiling-view, static HGR.

#### 3.1.2. Custom Ceiling Dataset (CeilGest)

To enable systematic evaluation of the frontal-to-ceiling domain gap, we collected and manually annotated CeilGest, a ceiling-view hand gesture dataset comprising 156,282 RGB images from 68 participants (41 male, 27 female; ages 19–58 years, median 26). All images were acquired using cameras mounted 2.5–3.0 m above floor level, closely reflecting the installation geometry of ceiling-mounted smart-home cameras. Unlike existing HGR datasets, which predominantly assume frontal or egocentric viewpoints, CeilGest captures the overhead perspective encountered in practical smart-home environments while preserving the same 18 static gesture vocabulary used by HaGRID. Recordings were carried out in the participants’ own homes, so the dataset spans 68 distinct domestic environments—one per participant—rather than a single instrumented room or laboratory setting. Because acquisition took place in real dwellings, several factors varied without experimental control: ceiling height within the 2.5–3.0 m mounting range, illumination (daylight and artificial, unregulated), room geometry and furniture layout, and consequently the subject-to-camera distance and viewing angle. These constitute nuisance variation spanned by the dataset rather than experimental conditions; their individual contributions were not isolated, and no controlled distance or illumination study was conducted.

Each participant performed all 18 gesture classes multiple times. Rather than recording a single continuous pose, every gesture was repeated approximately five times (varying across participants). Between repetitions, the participant completely lowered the hand before re-performing the gesture, introducing natural variations in hand position, in-plane orientation, wrist pose, and approach angle. Each repetition was held for approximately 5 s.

**Figure 1:**
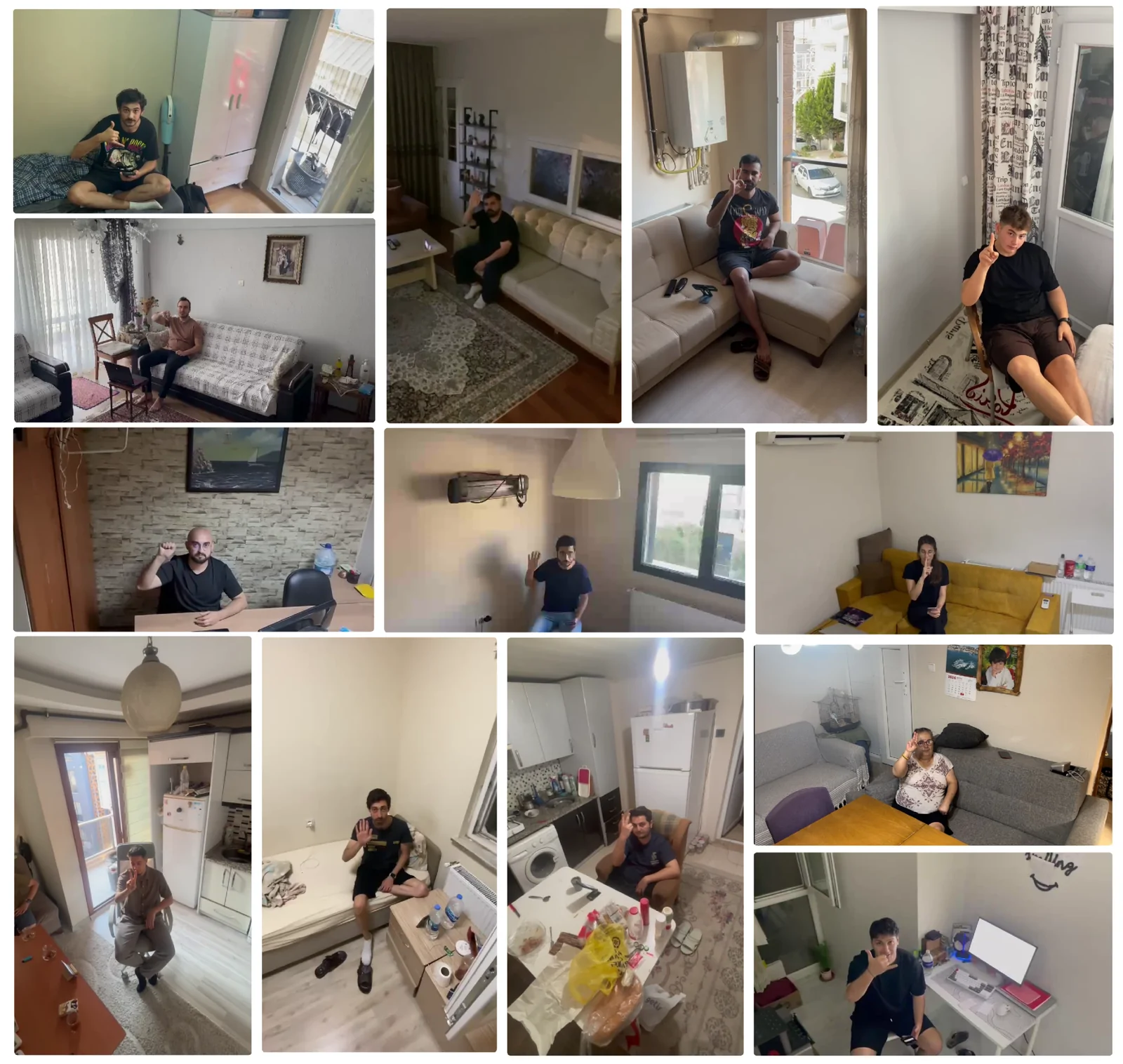
Samples from the proposed CeilGest dataset. The examples illustrate the diversity intentionally captured during data acquisition, including 68 distinct domestic environments (one per participant), illumination conditions, backgrounds, subject-to-camera distances, and viewing geometries representative of ceiling-mounted smart-home cameras. The figure also highlights the perspective distortion and hand-orientation variations characteristic of overhead imaging, which distinguish CeilGest from conventional frontal-view gesture datasets and motivate the frontal-to-ceiling domain-gap analysis presented in this work. All participants provided written informed consent.

All sessions were recorded at 30 fps with a resolution of 1280×720 px. To reduce redundancy while preserving temporal diversity, frames were sampled at 5 fps, yielding approximately 2300 manually annotated images per participant. Representative frames illustrating the range of environments, illumination conditions and viewing geometries are shown in Figure 1.

Although consecutive frames within an individual repetition remain visually similar, the repeated lowering and re-positioning of the hand means that each (participant, gesture) pair contributes multiple distinct pose realisations rather than a single sustained static observation. Consequently, CeilGest provides substantially greater appearance variability than would be obtained from one sustained gesture per participant. Nevertheless, temporal correlation remains within each repetition, and multiple repetitions from the same participant should not be regarded as fully independent statistical observations, as discussed in Section 5.4.

For the held-out test split specifically, each (subject, gesture) instance comprises approximately five separate repetitions, with the hand re-posed between takes. This yields approximately 50 separate gesture repetitions per class (10 held-out subjects × ≈5 repetitions), compared with 1500 sampled frames per class. These repetitions provide a more appropriate scale for considering temporal dependence than the raw frame count, but they are not fully independent because multiple repetitions originate from the same 10 held-out subjects. Consequently, generalisation to unseen individuals remains primarily constrained by the number and diversity of held-out subjects. This issue is quantified further in Section 4.3 and discussed as a limitation in Section 5.4.

Data were split at the subject level to prevent identity leakage: 50 subjects (73.5%) for training, 8 (11.8%) for validation, and 10 (14.7%) for testing. Because each participant was recorded in a different home, this split is simultaneously environment-disjoint: the ten test subjects were recorded in ten domestic environments appearing in neither the training nor the validation split. Reported test performance therefore reflects generalisation to unseen individuals in unseen rooms, rather than to unseen individuals within a shared recording environment. The test set comprises 27,000 images (1500 per class) from held-out subjects only.

### 3.2. System Architecture and Edge Deployment

The system follows four design principles: *privacy by design* (all processing occurs locally), *real-time responsiveness*, *robustness* (temporal verification filters incidental movements), and *broad coverage* (ceiling mounting provides unobstructed room coverage, a configuration that is also relevant for users with limited mobility).

**Figure 2:**
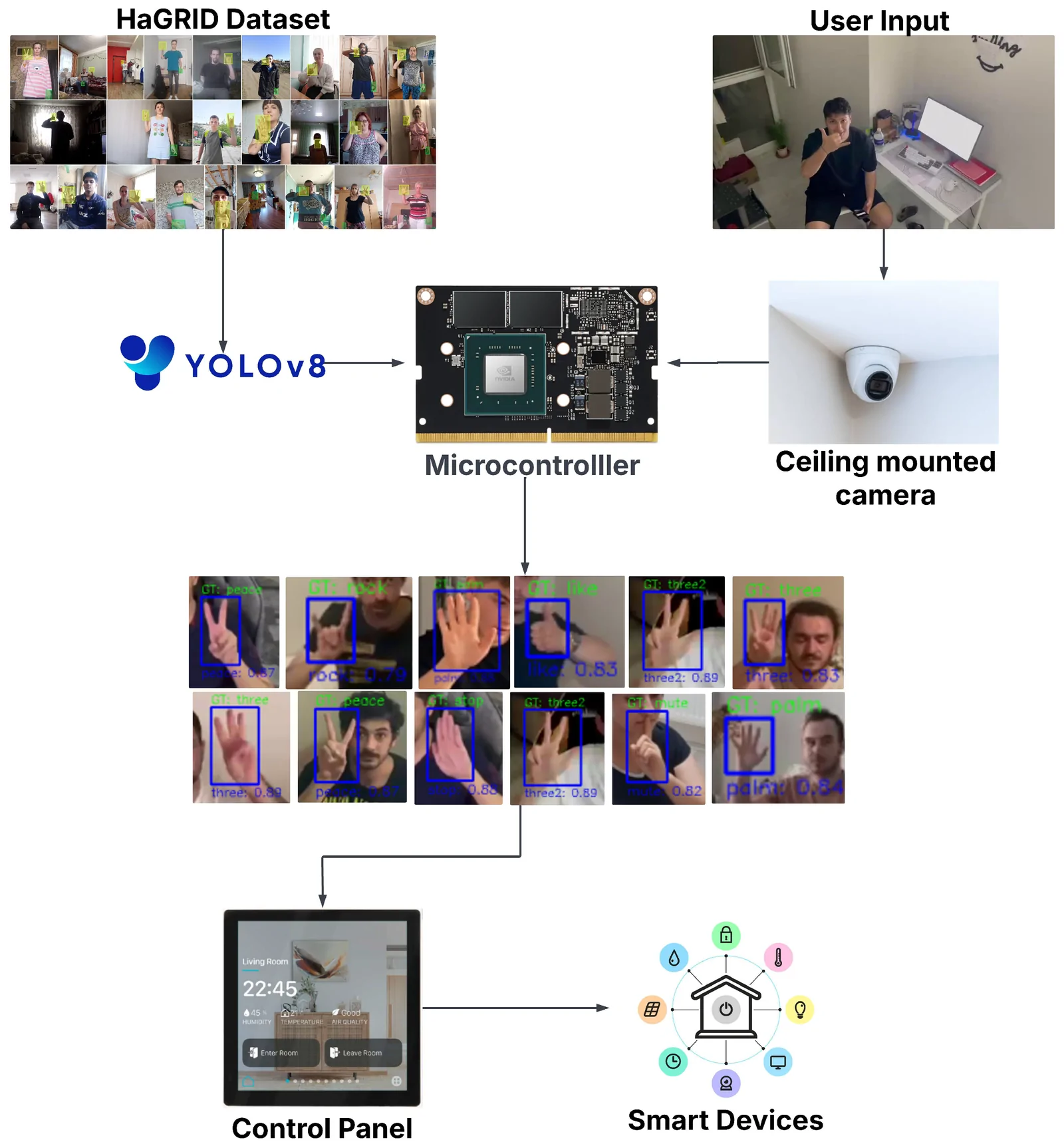
End-to-end system architecture for ceiling-mounted HGR. A YOLOv8n model, initialised from COCO-pretrained weights and trained on CeilGest, runs on an edge device (Raspberry Pi 5 via NCNN or Jetson Orin Nano via TensorRT). The camera is mounted 2.5–3.0 m above floor level and captures 1280×720 px frames at 30 fps. Detected gestures are temporally verified over a 2 s sliding window (confidence threshold τ=0.7) before a UART command is transmitted to the appliance controller, which triggers the corresponding smart-home action and provides auditory feedback. Detection examples (centre) illustrate bounding-box outputs with class labels and confidence scores.

The end-to-end pipeline (Figure 2) comprises six stages: (1) image acquisition from a ceiling-mounted camera at 2.5–3.0 m above floor level (1280×720 px, 30 fps); (2) CPU-side preprocessing (letterbox resize to 640×640 px, normalisation to [0,1] in RGB); (3) gesture detection via YOLOv8n with platform-specific backends (Section 3.5); (4) temporal verification requiring consistent classification over a 2 s sliding window with confidence threshold τ=0.7 (Section 3.2); (5) UART command transmission at 115,200 baud (8N1) to appliance controllers; and (6) auditory feedback confirming each action via a 0.5 s audio clip.

Per-frame inference latency on both platforms is well below one second (see Section 4), enabling real-time visual feedback. The 2 s temporal verification window introduces an intentional activation delay to suppress false positives; consequently, the end-to-end time from the onset of a sustained gesture to appliance actuation is approximately 2.1–2.4 s depending on the platform. We consider this acceptable for non-urgent smart-home commands, where reliability takes precedence over instantaneous response.

Two inference configurations are evaluated.

- **RPi 5 (CPU):** NCNN backend with ARM NEON single-instruction-multiple-data (SIMD) vectorisation (FP32).
- **Jetson Orin Nano:** TensorRT backend with layer fusion, kernel auto-tuning, and FP16 Tensor Core acceleration.

These two platforms differ principally in computation and memory. The Raspberry Pi 5 (Raspberry Pi Ltd., Cambridge, UK) uses a BCM2712 SoC (quad-core Cortex-A76, 4 GB LPDDR4X, ≈12 W) with the NCNN backend (FP32, ARM NEON) and a PiCamera V3 (CSI; Raspberry Pi Ltd., Cambridge, UK), while the Jetson Orin Nano (NVIDIA Corporation, Santa Clara, CA, USA) uses an 8 GB Orin module (6-core Cortex-A78AE, 1024-core Ampere GPU, 15 W) on a Leetop A603 carrier board (Leetop Technology, Shenzhen, China) with TensorRT 10.3.0 (FP16, JetPack 6.2.1) and a USB webcam. The software environments were Debian 12 (bookworm, 64-bit) with Python 3.11.2, OpenCV 4.8.0 and NumPy 1.24.3 on the Raspberry Pi 5, and Ubuntu 22.04.5 LTS with Python 3.10.12, OpenCV 4.12.0, NumPy 2.2.6 and CUDA 12.6.68 on the Jetson Orin Nano.

Recognised gestures are mapped to single-byte UART commands transmitted at 115,200 baud (8N1) via an FT232R USB-to-TTL converter (FTDI Ltd., Glasgow, UK). The receiving Arduino Nano (Arduino S.r.l., Monza, Italy) acknowledges each command with a single-byte ACK (0xFF) within 50 ms; unacknowledged commands are retransmitted once. The complete inference loop, including temporal verification and command transmission, is summarised in Algorithm 1.
**Algorithm 1** End-to-end ceiling-view HGR inference pipeline (per device)**Require:** 
Ceiling camera stream (1280×720, 30 fps); exported engine *net* for backend *B* ∈ {ncnn, TensorRT}; gesture-to-command map cmd[·]**Require:** 
τ=0.7, α=0.80, β=0.90, $T_{w}=2.0$ s, $T_{r}=1.0$ s, $M_{\min}=2$  1: *net* ← LoadEngine(*B*) ▹ NCNN FP32 (ARM NEON) or TensorRT FP16  2: W←∅ ▹ sliding window of triples $\left(t_{i},{\hat{y}}_{i},p_{i}\right)$  3: $t_{\mathrm{last}}\leftarrow -\infty$ ▹ time of last confirmed command  4: **while** camera active **do**
  5:        $I_{\mathrm{raw}},t\leftarrow$Acquire
  6:        *I* ← Letterbox$\left(I_{\mathrm{raw}},640\times 640\right)$
  7:        I←I/255 **in** RGB ▹ normalisation to [0,1]  8:        *D* ← *net*.Infer(I) ▹ boxes, classes, confidences  9:        **if** *B* requires NMS **then**
10:                D←NMS(D,IoU=0.7) ▹ skipped for NMS-free YOLOv1011:        **end if**
12:        **if** D=∅ **then**
13:                $\left({\hat{y}}_{t},p_{t}\right)\leftarrow \left(\mathrm{no}\_\mathrm{gesture},0\right)$
14:        **else**
15:                $\left({\hat{y}}_{t},p_{t}\right)\leftarrow \arg\max_{d\in D}p_{d}$ ▹ highest-confidence detection16:        **end if**
17:        $W\leftarrow W\cup \{\left(t,{\hat{y}}_{t},p_{t}\right)\}$
18:        $W\leftarrow \{\left(t_{i},{\hat{y}}_{i},p_{i}\right)\in W:t-t_{i}\leq T_{w}\}$ ▹ trailing-interval window; $\left|W\right|=M_{t}$19:        **if** $t-t_{\mathrm{last}}\geq T_{r}$ **then**▹ refractory period elapsed20:                $\hat{c}$ ← TemporalVerify(W,t)
21:                **if** $\hat{c}\neq$
null **then**
22:                      SendCommand($\mathrm{cmd}[\hat{c}]$)
23:                      PlayFeedback($\hat{c}$) ▹ 0.5 s audio confirmation24:                      W←∅;    $t_{\mathrm{last}}\leftarrow t$ ▹ NCNN FP32 (ARM NEON) or TensorRT FP1625:                **end if**
26:        **end if**
27:        **discard** $I_{\mathrm{raw}},I,D$ ▹ no frame stored or transmitted28: **end while**


29: **procedure** SendCommand(*b*)
30:        UartWrite(*b*) ▹ 115,200 baud, 8N131:        **if not** AwaitAck(0xFF, 50 ms) **then**
32:                UartWrite(*b*) ▹ single retransmission33:        **end if**
34: **end procedure**


#### Temporal Verification

Rather than triggering actions on single-frame detections, the system requires consistent recognition over a sliding window to distinguish intentional gestures from incidental hand movements. At each frame *t*, the detector produces a class prediction ${\hat{y}}_{t}$ with confidence $p_{t}$. A detection is considered *valid* if $p_{t}\geq \tau$ and ${\hat{y}}_{t}\neq \mathrm{no}\_\mathrm{gesture}$, where τ=0.7.

Let $W_{t}$ denote the sliding window containing all frames acquired during the trailing interval $\left[t-T_{w},\;t\right]$, where $T_{w}=2.0$ s is the window duration. Unlike a fixed-length frame buffer, $W_{t}$ adapts to the achieved frame rate: its cardinality $|W_{t}|=M_{t}$ equals the number of frames the device actually processes within $T_{w}$, so the criterion is independent of inference throughput. Let $V_{t}\subseteq W_{t}$ be the subset of valid detections within the window, and let ${\hat{c}}_{t}=\mathrm{mode}\left\{{\hat{y}}_{i}:i\in V_{t}\right\}$ be the modal class among valid frames.

A gesture is *confirmed* at time *t* if and only if the window spans the full duration $T_{w}$, contains at least a minimum number of processed frames ($M_{t}\geq M_{\min}$, with $M_{\min}=2$ to guarantee a meaningful vote at low frame rates), and the following two conditions are jointly satisfied:

1. **Confidence gate (fraction-based):** $|V_{t}|/M_{t}\;\geq \;\alpha$, where α=0.80; i.e., at least 80% of the frames processed within the window carry a valid detection above threshold.
2. **Class consistency:** $|\left\{i\in V_{t}:{\hat{y}}_{i}={\hat{c}}_{t}\right\}\left|\;/\;\right|V_{t}|\;\geq \;\beta$, where β=0.90; i.e., at least 90% of the valid frames agree on the same gesture class.

Expressing both gates as fractions of the frames actually processed—rather than as fixed counts derived from a nominal 30 fps—makes the verification logic invariant to the deployment frame rate: the same α,β thresholds apply whether the device sustains 30 fps or the ∼6 fps observed on the Raspberry Pi 5.

Upon confirmation, the modal class ${\hat{c}}_{t}$ is emitted as a UART command and the window buffer is cleared to prevent repeated triggering. A refractory period of $T_{r}=1.0$ s is imposed before the next gesture can be confirmed. The verification procedure is given in Algorithm 2.

The temporal-verification parameters (τ=0.7, α=0.80, β=0.90, $T_{w}=2.0$ s, and $T_{r}=1.0$ s) were selected empirically using the CeilGest validation experiments. The confidence threshold τ was additionally assessed through the F1–confidence analysis described in Section 4.5. The selected values of α and β correspond to requiring high proportions of valid and class-consistent detections within each verification window, while $T_{w}$ and $T_{r}$ define the verification and refractory intervals, respectively.

Three properties of this parameterisation are worth making explicit, since they determine how sensitive the verification stage is to the particular values chosen. First, the detection threshold τ sits well inside a region over which the in-domain detector is insensitive: the F1–confidence characterisation of Section 4.5 shows F1 >0.95 maintained across the range 0.10–0.70, with a peak at 0.60, so τ=0.7 lies at the upper edge of a broad plateau rather than at a sharp optimum. It is the flatness of that curve, not the absolute level of F1, that carries the argument: the threshold sweep is a paired comparison on identical frames, so the sample-size inflation discussed in Section 4.3 affects every operating point equally and cancels in the shape, even though it leaves the absolute value imprecise. The frontal-trained models peak at lower thresholds (0.40–0.45), which is itself a symptom of the domain shift rather than a property of the verification rule.

Second, α and β are more readily interpreted as frame budgets than as abstract fractions. At the deployment frame rate of the Raspberry Pi 5 (∼6 fps; Section 4.6) the window contains $M_{t}\approx 12$ processed frames, so α=0.80 permits at most two frames within the window to yield no valid detection, and β=0.90 permits at most one of the remaining valid frames to disagree with the modal class. At the full camera rate the same fractions correspond to twelve and five frames, respectively. Expressing the gates as fractions rather than counts is what keeps this interpretation stable as throughput varies—including under the thermal degradation reported in Section 4.6, which reduces $M_{t}$ but leaves the admissible error budget proportionally unchanged.

Third, the 2 s verification window $T_{w}$ defines the nominal observation interval before a gesture can be confirmed, whereas τ, α, and β influence whether the activation criterion is satisfied within that interval. More stringent thresholds may therefore increase the realised activation latency by causing additional windows to be rejected. The refractory period $T_{r}$ constrains the timing of subsequent activations rather than the initial confirmation delay. A full factorial sweep of τ, α, and β against measured false-activation rate and end-to-end activation latency was not performed and is identified as a limitation in Section 5.4.
**Algorithm 2** Temporal verification over the sliding window $W_{t}$  1: **procedure** TemporalVerify(W,t)
  2:        **if** $t-\min\left\{t_{i}:i\in W\right\}<T_{w}$ **or** $|W|<M_{\min}$ **then**
  3:                **return** null
▹ window not yet full/too few frames
  4:        **end if**
  5:        $M_{t}\leftarrow \left|W\right|$ ▹ frames actually processed within $T_{w}$
  6:        $V_{t}\leftarrow \left\{i\in W:p_{i}\geq \tau \;\wedge \;{\hat{y}}_{i}\neq \mathrm{no}\_\mathrm{gesture}\right\}$  7:        **if** $|V_{t}|/M_{t}<\alpha$ **then**  8:                **return** null▹ confidence gate (fraction-based)  9:        **end if**10:        ${\hat{c}}_{t}\leftarrow \mathrm{mode}\left\{{\hat{y}}_{i}:i\in V_{t}\right\}$11:        **if** $|\left\{i\in V_{t}:{\hat{y}}_{i}={\hat{c}}_{t}\right\}\left|/\right|V_{t}|<\beta$ **then**12:                **return** null
▹ class-consistency criterion13:        **end if**14:        **return** ${\hat{c}}_{t}$
▹ gesture confirmed15: **end procedure**


### 3.3. YOLO Architectures and Model Selection

YOLOv8 [7] is available in five scales (n/s/m/l/x) employing anchor-free detection, a CSPDarknet backbone, C2f feature fusion modules, and a decoupled detection head. The anchor-free formulation improves robustness to the variable hand sizes encountered at different distances from the ceiling camera, and C2f modules enhance gradient flow at minimal computational cost. We evaluate all five variants under identical training conditions (Section 3.4) to determine whether gesture recognition benefits from increased model capacity.

For cross-generation comparison, we additionally evaluate YOLOv10n and YOLOv10x [8], which introduce NMS-free detection via consistent dual assignments. YOLOv10n (2.78 M parameters, 8.7 GFLOPs) is the smallest model in our evaluation; YOLOv10x (31.81 M, 171.8 GFLOPs) is most directly comparable to YOLOv8l (43.69 M, 165.7 GFLOPs) in compute but requires substantially fewer parameters. Only the two endpoints are evaluated to bracket the generation’s range. This design serves two purposes: the YOLOv8 sweep reveals whether model capacity impacts performance, and the YOLOv10 endpoints assess whether NMS-free inference provides tangible benefits for edge-deployed HGR.

We note that YOLOv11 and subsequent iterations (v12, v13) were released during the preparation of this manuscript. Gao et al. [9] report competitive HGR accuracy for these architectures on frontal-view data. These models were not included in our evaluation due to time and computational resource constraints. Our primary research question—whether the frontal-to-ceiling domain shift degrades detection performance, and whether model capacity affects this—is fully addressable within the YOLOv8/v10 design space, which spans a representative range from 2.78 M to 68.23 M parameters.

### 3.4. Training Procedure

#### 3.4.1. HaGRID Training (All Variants)

All variants were trained on HaGRID under identical hyperparameters: SGD with initial learning rate 0.01, momentum 0.9, weight decay $5\times 10^{-4}$, and linear warmup over 3 epochs. Mixed-precision (FP16) training was enabled throughout. Images were resized to 640×640 px.

Augmentation comprised RandAugment (N=2 transforms per image, magnitude M=9 on the Ultralytics scale), horizontal flipping (p=0.5), HSV jittering (hue ± 0.015, saturation ± 0.7, value ± 0.4), Gaussian blur (σ∈[0.1,2.0], p=0.1), and CLAHE (clip limit = 4.0, tile grid 8×8, p=0.1). Mosaic augmentation was enabled with probability 1.0 for the first 90% of training epochs and disabled thereafter, following the default Ultralytics schedule.

The composite loss comprised box regression, classification, and distribution focal loss (DFL) terms (weights: 7.5, 0.5, 1.5). No layers were frozen; all parameters were updated from COCO-pretrained initialisations.

All variants were trained for a fixed budget of 25 epochs. Convergence was monitored during training by inspecting validation mAP_50_ at each epoch; all variants exhibited diminishing improvement in the final five epochs, with epoch-over-epoch gains below 0.2 percentage points. To partially address this limitation, we note that final validation mAP_50_ values for all variants are consistent with or exceed published benchmarks on HaGRID at comparable epoch counts [9], suggesting adequate convergence.

Batch size was 16 for all variants except YOLOv8l and YOLOv8x, which used 8 due to GPU memory constraints. We acknowledge that reducing the batch size from 16 to 8 for these two variants alters the effective learning rate and gradient-noise characteristics and may introduce minor differences in convergence behaviour. Given the metric ceiling observed across all variants (Section 4.2), we do not expect this to affect the conclusions drawn from these experiments.

#### 3.4.2. CeilGest Dataset Training

To evaluate detection performance under the overhead perspective, all model variants were initialised from COCO-pretrained weights and trained on CeilGest (Section 3.1.2)—that is, they were not fine-tuned from the HaGRID-trained checkpoints. This design choice isolates the effect of the ceiling-view training data from any residual frontal-domain bias that HaGRID fine-tuning might carry, providing a conservative baseline for the overhead deployment scenario.

Training hyperparameters were identical to those in Section 3.4.1 (SGD, lr = 0.01, momentum = 0.9, weight decay = $5\times 10^{-4}$, 640×640 px input, FP16 mixed precision). As with the HaGRID experiments, batch size was 16 for all variants except YOLOv8l and YOLOv8x (batch size 8).

### 3.5. Model Export and Quantisation

Trained PyTorch weights are exported through a three-stage pipeline:

**Stage 1 (ONNX export):** Weights are exported to ONNX (opset 17) via the Ultralytics API and simplified using ONNX-Simplifier [41] (v0.4.35) to remove redundant operations without altering numerical output.

**Stage 2 (Platform-specific conversion): NCNN (RPi 5 CPU):** Converted via onnx2ncnn; inference uses ARM NEON SIMD in FP32. **TensorRT (Jetson):** Converted to a TensorRT engine (v10.3.0) at FP16 precision with automatic layer fusion, kernel auto-tuning, and Tensor Core scheduling.

## 4. Experimental Results

This section presents classification baselines (Section 4.1), YOLO capacity analysis on frontal data (Section 4.2), ceiling-mounted evaluation with domain gap analysis (Section 4.3), edge deployment validation (Section 4.6), and a consolidated findings summary (Section 4.7).

### 4.1. Classification Baselines and Model Selection

Prior to applying the detection framework to CeilGest, a classification analysis on HaGRID was conducted to evaluate various benchmark models (Table 2). Five models were trained and tested under identical conditions (AdamW, batch 32, He initialisation, 25 epochs, 224×224 px, augmentation with colour jitter, grid distortion, elastic transformation) and evaluated on a balanced 90,000-image test set (5000 per class).

YOLOv8n (0.9960) achieved the highest accuracy despite being almost 3× smaller than the second most accurate model: MobileNetV3Large (0.9905). The performance gap with ResNet18 (0.9800) is distinguishable at the test-sampling level, whereas the gap between MobileNetV3Large and EfficientNetV2 lies within overlapping intervals and should be considered marginal.

A colour space comparison (Table 2) revealed negligible variation with RGB yielding the highest accuracy. These baselines indicate that colour space choice (<0.7 percentage points) has a smaller effect than model architecture (≈1%), both of which are comparable to or smaller than the domain-shift effect (up to 5.5 percentage points of per-class AP on the worst-affected class, alongside a ≈24× increase in misclassifications; Section 4.4). This observation, combined with the advantages of single-stage detection (simultaneous localisation/recognition, temporal verification compatibility, lower latency), motivated the use of YOLOv8 variants evaluated in Table 3.

### 4.2. Model Capacity Analysis on HaGRID

Table 3 presents all YOLOv8 variants trained on HaGRID under similar conditions. The results reveal a metric ceiling effect at mAP_50_: all five variants—from nano (3.16 M parameters) to extra-large (68.23 M)—achieve identical mean mAP_50_ of 0.995. Differences emerge only at mAP_50–95_, ranging from 0.897 (YOLOv8n) to 0.908 (YOLOv8x)—a marginal 0.011 gain across 21.6× capacity increase.

This ceiling is partly a consequence of the lenient IoU = 0.50 threshold: at mAP_50–95_, a modest gap (0.897–0.908) shows larger models learn tighter bounding-box regression—negligible for gesture classification but potentially relevant for precise localisation. Since the nano variant matches at mAP_50_, the nano variant can be selected for edge deployment, reducing model size by 21× and computation by 29× relative to YOLOv8x. Batch size invariance (mAP_50_ fixed at 0.995, mAP_50–95_ varying by at most 0.002) is confirmed for the nano and small variants; larger variants could not be evaluated at higher batch sizes due to GPU memory constraints.

### 4.3. CeilGest Evaluation

Four models were evaluated on the CeilGest test set comprising 10 subjects held out through subject-level splitting (Section 3.1.2), using 27,000 images across 18 gesture classes (excluding no_gesture). The models span domain-specific training and architecture/capacity: (1) YOLOv8n_ceil_ (CeilGest-trained), (2) YOLOv8n_HaGRID_ (HaGRID-trained), (3) YOLOv10n_HaGRID_ (HaGRID-trained), and (4) YOLOv10x_HaGRID_ (31.81 M params, HaGRID-trained).

The CeilGest test set comprises 27,000 frames (1500 per class) from 10 held-out subjects, and these frames are strongly temporally correlated: each subject contributed approximately five separate ≈5 s holds per gesture, with the hand lowered and re-posed between takes. Frames within an individual hold therefore represent repeated observations of essentially the same gesture instance. At the gesture-repetition level, the test set contains approximately 50 separate holds per class, far fewer than the 1500 sampled frames. Moreover, these holds remain clustered within only 10 subjects and should not themselves be treated as fully independent observations. Accordingly, the per-frame metrics reported in this section should primarily be interpreted as measures of detection consistency on the held-out ceiling-view recordings.

To examine the sensitivity of the error analysis to within-hold temporal dependence, we translated the frame-level error counts reported in Section 4.4 into approximate hold-level quantities. The test split contains approximately 900 gesture holds (10 subjects × 18 gestures × ≈5 repetitions). Given 27,000 test frames, this corresponds empirically to approximately 30 sampled frames per hold on average. This empirically derived value is used for the analytical sensitivity analysis below (Table 4).

We stress that these are analytical bounds derived from the frame-level counts already reported, not a separate empirical evaluation; they rest on the assumption of an approximately uniform 30 frames per hold. Two observations follow. First, both the E/30 estimate and the conservative majority-vote bound indicate a substantially larger number of potentially affected holds for the frontal-trained model than for the in-domain model. This supports the conclusion that the principal domain-gap finding is not dependent on treating all 27,000 frames as statistically independent observations. However, because these quantities are analytical estimates derived from frame-level error counts rather than direct hold-level re-aggregation, they should not be interpreted as an independent estimate of the exact error ratio.

Second, accounting for the gesture-repetition structure substantially increases the uncertainty associated with class-specific error estimates. For the worst-affected class, call under YOLOv8n_HaGRID_, the frame-level error rate was 139/1500=9.3%. The corresponding hold-level sensitivity analysis indicates considerably greater uncertainty than the frame count alone would suggest. Thus, the raw number of frames overstates the statistical precision of the evaluation, and generalisation to unseen individuals remains constrained by the 10 held-out subjects. Reporting gesture-level performance at this resolution would require a larger number of held-out subjects and repetitions, which we identify as a priority for future data collection (Section 5.5).

Table 5 presents the results for the models listed above. These values should be read with the effective sample size in mind. In particular, absolute figures near the top of the scale and small differences in the second or third decimal place should not be interpreted as meaningful model rankings given the effective number of gesture repetitions and the clustering of repetitions within subjects.

To make this distinction explicit, we separate the quantities reported for the ceiling test set into two groups. The caveat is specific to this evaluation: the HaGRID results of Section 4.1 and Section 4.2 are computed over 90,000 images from tens of thousands of contributors and are not affected by the correlation discussed here. *Quantities we regard as reliable for comparison* are large, paired differences measured on identical test footage—in particular the ratio of class-to-class misclassification counts between models, and per-class AP drops of several percentage points. Because every model is evaluated on the same frames, the paired design controls for differences in test-set composition and substantially strengthens the between-model comparison, although temporal correlation still limits the statistical precision of the absolute estimates. *Quantities we regard as unreliable for comparison* are the absolute values of aggregate detection metrics (mAP_50_, mAP_50–95_, precision, recall, F1) and any between-model difference in their second or third decimal place. Readers should not infer a performance ordering from such differences, and none of the conclusions of this work rests on them.

One third-decimal difference is worth explaining rather than ranking. The precision of YOLOv8n_ceil_ (0.979) is nominally below that of YOLOv10x_HaGRID_ (0.981); by the criterion just stated this difference is too small to support an ordering, but its *origin* is informative and specific to the overhead view. It stems from duplicate detections: the model occasionally produces overlapping boxes for a single hand, with near-duplicates surviving NMS (IoU 0.7)—particularly where the wrist and forearm create elongated regions from overhead. These are counted as false positives in evaluation but are filtered by temporal verification in practice, so operational precision exceeds the per-frame metric.

### 4.4. Domain Gap Analysis

**Figure 3:**
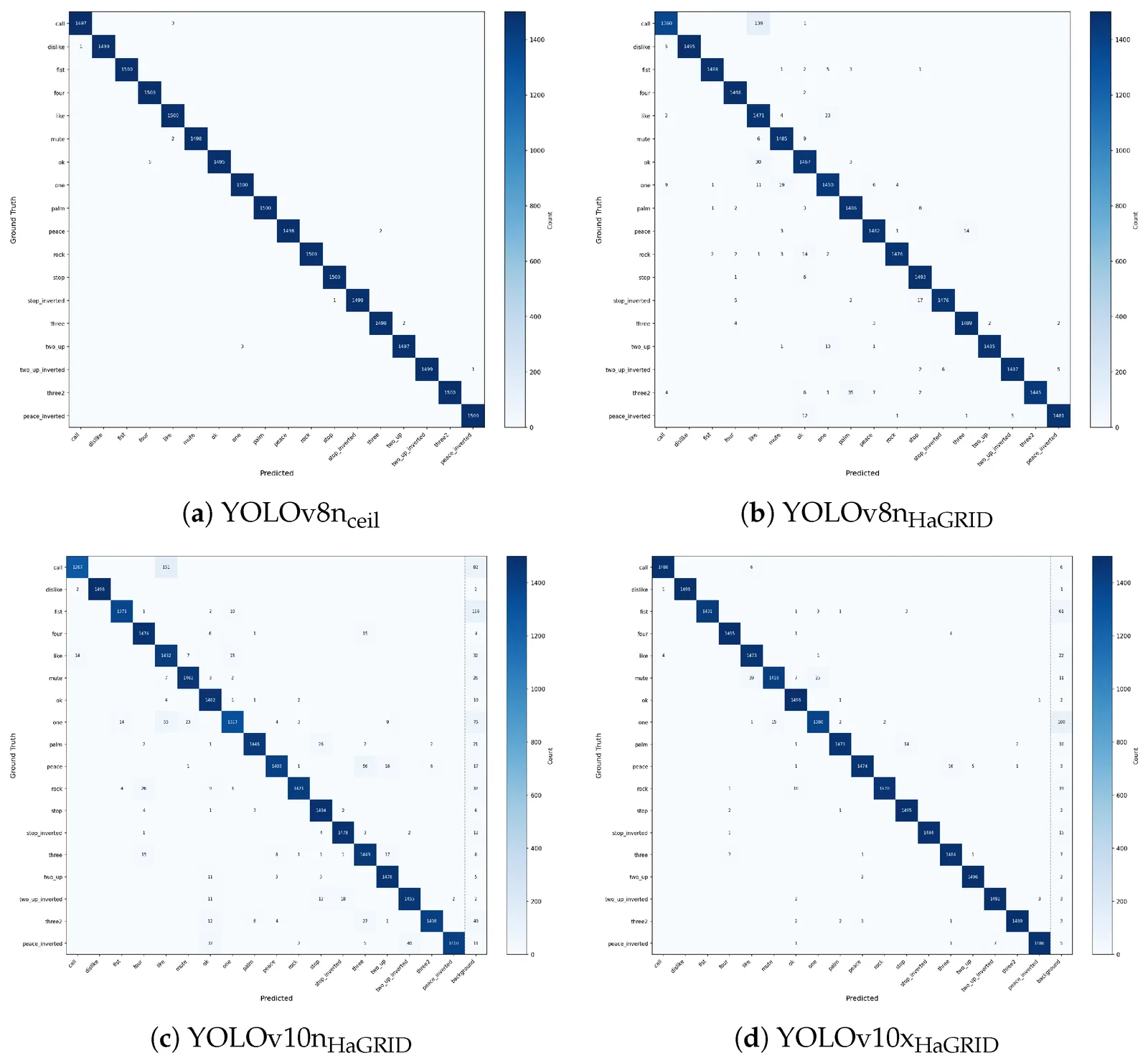
Confusion matrices for all four models on the 18-class ceiling test set. The domain-trained model (**a**) is near-ideal diagonal; HaGRID-trained models (**b**–**d**) show degradation concentrated on call, one, and like.

The frontal-to-ceiling domain gap was evaluated by applying HaGRID-trained models to the CeilGest test set and comparing their performance with the in-domain CeilGest-trained YOLOv8n model. Table 6 summarises the resulting degradation on the ceiling-view test set, while Figure 3 provides the corresponding class-level confusion matrices.

The clearest effect of the domain shift is observed for YOLOv8n. On the same 27,000 ceiling-view frames, the HaGRID-trained YOLOv8n produced 486 gesture misclassifications, compared with only 20 for the CeilGest-trained YOLOv8n, corresponding to approximately a 24× increase in errors when training and deployment viewpoints are mismatched. Because both models were evaluated on identical test frames, this paired comparison is not affected by differences in test-set composition.

Increasing model capacity partially compensated for the viewpoint mismatch but did not eliminate it. The larger HaGRID-trained YOLOv10x reduced total misclassifications to 206, compared with 761 for YOLOv10n and 486 for YOLOv8n. Nevertheless, the much smaller CeilGest-trained YOLOv8n produced only 20 misclassifications. Thus, among the configurations evaluated, matching the training viewpoint to the deployment viewpoint produced a substantially greater reduction in errors than increasing model capacity.

The degradation was strongly class-dependent. The most affected gesture was call, for which AP decreased from approximately 1.000 under the frontal HaGRID condition to 0.945 when YOLOv8n_HaGRID_ was evaluated on ceiling-view data, corresponding to a 5.5-percentage-point decrease. The dominant confusion was call→like: 139 of 1500 call frames (9.3%) were classified as like. Other recurrent errors included three2→palm and peace→three. These patterns indicate that the domain shift particularly affects gestures distinguished by finger orientation and configuration, consistent with the geometric effects of the overhead viewpoint discussed in Section 5.

Total class-to-class misclassifications across the 27,000-frame test set were 20 (YOLOv8n_ceil_), 486 (YOLOv8n_HaGRID_), 761 (YOLOv10n_HaGRID_), and 206 (YOLOv10x_HaGRID_). Two patterns emerge. First, model capacity *does* reduce the gap: the 31.81 M-parameter YOLOv10x_HaGRID_ handles the overhead view considerably better than the nano models—call→like drops to 6 samples and total errors to 206, under a third of the same-architecture YOLOv10n_HaGRID_’s 761—so capacity is not irrelevant under domain shift, although this comes at a substantially higher computational cost: YOLOv10x carries 11× the parameters and roughly 20× the FLOPs of the nano model (Section 3.3). Second, in-domain training nonetheless remains decisively superior: the 3.16 M-parameter YOLOv8n_ceil_ reaches 20 errors, more than an order of magnitude below the best frontal-trained model (206) despite using ≈10× fewer parameters than YOLOv10x_HaGRID_.

At comparable nano scale, YOLOv10n_HaGRID_ produced more ceiling-view errors than YOLOv8n_HaGRID_. This difference may reflect model capacity, architectural design (including YOLOv10’s NMS-free dual-assignment strategy) or their interaction. Because the present experiments do not isolate these factors, no causal attribution can be made. Establishing the specific contribution of NMS-free inference would require a controlled comparison between matched-capacity NMS-based and NMS-free models. Accordingly, the architecture-related observation in Table 7 should be interpreted as qualitative engineering guidance rather than evidence of a specific causal mechanism.

Combining these results with the classification baselines of Section 4.1 suggests an indicative heuristic for deployment engineering summarised in Table 7.

Among these factors, domain-matched training data produced the largest improvement relative to no adaptation. The YOLOv8n_ceil_ was trained on ceiling data from COCO-pretrained weights rather than fine-tuned from the HaGRID-trained checkpoint; the relative contribution of in-domain data versus the choice of initialisation therefore cannot be fully isolated. Whether domain adaptation methods could achieve comparable gains without target-domain labels was not tested (Section 5.5); accordingly, this result establishes what domain-matched data can achieve rather than a comparative ranking against domain adaptation.

### 4.5. Detailed Model Analysis

Figure 3 provides a class-wise view of the error patterns underlying the aggregate results in Section 4.4. The YOLOv8n_ceil_ confusion matrix is nearly diagonal, whereas the HaGRID-trained models exhibit distinct off-diagonal patterns under the ceiling-view domain shift. For YOLOv8n_HaGRID_, recurrent confusions include call→like, three2→palm, stop_inverted→stop, and peace_inverted→ok. YOLOv10n_HaGRID_ exhibits the most diffuse error distribution, with one alone accumulating 108 misclassifications across six classes. In contrast, YOLOv10x_HaGRID_ produces a cleaner confusion matrix, with its largest residual confusions occurring for mute→like (39) and peace→three (16), indicating greater robustness to the evaluated viewpoint shift.

The class-wise AP results further show that aggregate mAP can obscure gesture-specific degradation. YOLOv8n_ceil_ achieves AP ≥0.999 for all 18 gesture classes. Among the HaGRID-trained nano models, call is particularly affected, with AP values of 0.945 for YOLOv8n_HaGRID_ and 0.979 for YOLOv10n_HaGRID_. YOLOv10x_HaGRID_ maintains AP ≥0.985 across all classes, with one as its lowest-performing class. These results demonstrate that the relatively small changes in aggregate mAP_50_ can conceal substantially larger degradation for individual gestures, supporting the need for class-wise evaluation when assessing viewpoint robustness. As discussed in Section 4.3, the near-uniform AP of the in-domain model should be interpreted as strong per-frame detection consistency rather than accuracy over independent observations.

F1–confidence analysis showed that YOLOv8n_ceil_ achieved a peak F1 of 0.998 at a confidence threshold of 0.60 and maintained F1 >0.95 over a broad threshold range (0.10–0.70), indicating limited sensitivity to threshold selection. This range is the empirical basis for the detection threshold τ=0.7 adopted in the temporal-verification stage (Section 3.2): the operating point lies within a plateau rather than at a sharp optimum, so the verification stage does not depend on a finely tuned value of τ. In contrast, the HaGRID-trained models reached their optimal F1 at lower thresholds (0.40–0.45), suggesting a shift in prediction-confidence characteristics when frontal-trained models were applied to ceiling-view data. YOLOv10n_HaGRID_ showed the greatest degradation at low confidence thresholds, consistent with its more diffuse error pattern in the confusion matrix.

### 4.6. Edge Deployment Validation

To evaluate the feasibility of fully on-device operation, the trained YOLO models were deployed on two low-power embedded platforms: Raspberry Pi 5 using the NCNN backend and NVIDIA Jetson Orin Nano using TensorRT. Both platforms processed live 1280×720 video from ceiling-mounted cameras, with model inference performed at 640×640 px.

The exported inference engines preserved the detection performance of their corresponding PyTorch checkpoints on the HaGRID-derived validation set. For YOLOv8n, the exported model achieved mAP_50_ = 0.995 and mAP_50–95_ = 0.897, matching the original checkpoint (Table 3). Thus, conversion to the hardware-specific inference backend introduced no measurable accuracy degradation at the validation level.

The complete pipeline was functionally validated on both platforms. Gestures captured from the ceiling-mounted camera were detected on-device, temporally verified, and translated into UART control signals for external devices. The 2 s temporal verification mechanism successfully suppressed transient predictions before command execution. During continuous operation, the Raspberry Pi 5 implementation displayed an observed processing rate of approximately 6.2 FPS, demonstrating that the pipeline can operate at a rate sufficient for the temporal verification mechanism.

All image processing was performed locally, and frames were discarded after inference without transmission to an external server, providing the privacy-preserving operation required for residential deployment.

#### Controlled Latency and Thermal Measurements

We carried out controlled measurements on both platforms under live operation with the ceiling-mounted camera, in order to characterise the behaviour that functional validation alone cannot establish. No recorded video or pre-existing image set was used; capture, pre-processing, inference and post-processing were timed separately on the live stream. The same exported checkpoint was driven through an identical pre-processing, decoding and NMS implementation on both devices, so that the only component differing between platforms was the inference call itself; the NCNN build was version 1.0.20250503. Both platforms were passively cooled, as in the intended deployment.

Capture in these runs used a single USB UVC camera at 640 × 480 on both devices rather than the 1280 × 720 acquisition of Section 3.2, so that capture behaviour was identical across platforms and any latency difference was attributable to the inference backend alone. Because inference operates on letterboxed 640 × 640 crops in either case, the inference-stage figures reported below are independent of capture resolution. The thermal load under the higher-resolution deployment capture would, if anything, be greater, so the degradation reported here should be read as a conservative estimate; on the Raspberry Pi the pipeline was in any case compute-limited rather than capture-limited, meaning the device was running continuously at capacity, which is the worst case for sustained thermal behaviour. Throughput figures from these runs are correspondingly not directly comparable with the end-to-end rate reported earlier in this section for the deployment configuration.

Inference latency was 12.72 ± 0.12 ms on the Jetson Orin Nano (TensorRT FP16; p50 12.68 ms, p95 12.94 ms) and 74.69 ± 4.72 ms on the Raspberry Pi 5 (NCNN FP32). The platforms differ not only in mean latency but also in stability: the standard deviation is 0.9% of the mean on the Jetson and 6.3% on the Raspberry Pi.

The Raspberry Pi’s Cortex-A76 cores processed the same model in 301.11 ms compared with 405.11 ms for the Jetson’s Cortex-A78AE CPU-only configuration. Thus, the substantially lower end-to-end inference latency observed for the Jetson in the accelerated configuration is primarily associated with GPU-accelerated TensorRT inference rather than superior CPU-only performance. On the Raspberry Pi, NCNN reduced inference latency by approximately 4.0× relative to PyTorch 2.13.0, further illustrating the importance of the inference backend on platforms without a dedicated accelerator.

The sharpest difference between the platforms is thermal. Over a ten-minute continuous run—a separate acquisition from the 60 s measurement reported above, begun from a warmer start, so that its first-minute mean exceeds the 74.69 ms of that shorter run—mean inference latency on the Raspberry Pi 5 rose from 76.69 ms in the first minute to 90.23 ms in the last, a degradation of 17.6%, while junction temperature reached 90.8 °C and the hardware raised its frequency-capping and throttling flags. The degradation was not monotonic: latency climbed steeply over the first two minutes, held a plateau near 84–85 ms for roughly three minutes, then entered a second rise from the sixth minute onwards that coincided with temperatures above 89 °C. Critically, the system had not reached a stable operating point within the measurement window, so even a ten-minute run does not yield the equilibrium value; the second-half mean of 88.91 ms should be treated as a more representative lower bound for deployment planning than any figure measured in the opening seconds. Under the same workload the Jetson Orin Nano rose by 0.5 °C over sixty seconds and raised no throttling flags, and its latency drift was −0.3%. Realised throughput on the Raspberry Pi over the same ten-minute run fell from 12.62 to 11.02 frames per second; as noted above these figures are specific to the 640 × 480 capture configuration and are not comparable with the end-to-end rate reported earlier for the deployment configuration. Short measurements therefore systematically overstate the sustained performance of a passively cooled Raspberry Pi 5 on this workload.

Power consumption was not instrumented, and INT8 inference was not evaluated; both remain limitations of the present study.

These measurements characterise latency, sustained throughput and thermal behaviour, but they do not constitute a full deployment-grade benchmark (Section 5.4).

### 4.7. Summary of Key Findings

Table 8 consolidates the principal findings of the experimental analyses together with their scope and main caveats. Three results are particularly relevant to the objectives of this study. First, viewpoint mismatch produced the most pronounced performance degradation observed, with approximately 24× more class-to-class misclassifications for frontal-only than in-domain training on identical ceiling footage. Second, increasing model capacity provided comparatively limited benefit: lightweight YOLOv8n achieved similar frontal-view mAP_50_ to substantially larger variants, while the in-domain YOLOv8n produced fewer ceiling-view errors than the larger frontal-trained models. Third, the complete HGR pipeline was deployed on both Raspberry Pi 5 (NCNN) and Jetson Orin Nano (TensorRT) and characterised under controlled conditions, establishing the feasibility of fully on-device operation while also identifying sustained thermal degradation on the passively cooled Raspberry Pi 5 as a deployment constraint.

These findings and their implications are discussed in the next section; the associated methodological limitations are consolidated in Section 5.4.

## 5. Discussion

Taken together, the results indicate that viewpoint mismatch is a key determinant of HGR performance under ceiling-mounted deployment, while increasing model capacity provides only partial compensation under the conditions evaluated. Viewpoint-matched training, by contrast, enables a lightweight detector to achieve substantially lower error rates and supports practical on-device deployment.

### 5.1. Viewpoint-Induced Domain Shift

The experiments demonstrate that viewpoint mismatch can substantially degrade HGR performance even when aggregate detection metrics remain high. The clearest evidence is the paired comparison on identical ceiling-view footage: YOLOv8n_HaGRID_ produced 486 class-to-class misclassifications, compared with only 20 for the in-domain YOLOv8n_ceil_, corresponding to an approximately 24× increase. Because both models were evaluated on the same test footage, this comparison is unaffected by differences in test-set composition and provides the most robust measure of the observed domain-shift effect. Importantly, the corresponding change in aggregate mAP_50_ is comparatively small, showing that performance metrics can conceal practically important class-specific failures.

The class-wise analysis further demonstrates this effect. The worst-affected gesture, call, exhibited an AP decrease of up to 5.5 percentage points under the frontal-to-ceiling shift. The resulting confusion patterns are geometrically interpretable. The call–like distinction depends strongly on thumb orientation, which becomes less discriminative under the overhead projection because of foreshortening and self-occlusion. Similarly, finger-count gestures such as one, peace, and three lose part of their inter-finger spread and orientation cues when fingers extend toward the camera rather than across the image plane. These observations are consistent with findings from 3D hand pose estimation, where out-of-distribution viewpoints have been shown to increase ambiguity through self-occlusion and loss of depth information [27,28].

Increasing model capacity partially reduced these cross-view errors but did not eliminate them. YOLOv10x_HaGRID_ produced a cleaner confusion matrix than the smaller frontal-trained models, suggesting that additional capacity can exploit residual cues such as wrist and forearm orientation. Nevertheless, the lightweight YOLOv8n_ceil_ produced substantially fewer errors than any frontal-trained model evaluated. Thus, within the configurations examined, matching the training and deployment viewpoints had a greater practical effect than increasing model capacity. This supports viewpoint-matched training data as an important consideration when HGR systems are deployed under camera geometries that differ substantially from those represented in conventional benchmarks.

The comparatively small effect observed for colour representation provides additional context for this finding: changes among the evaluated colour spaces produced only minor performance differences. We emphasise that subject-to-camera distance and illumination were not manipulated experimentally; they varied naturally across the 68 recording environments and are therefore spanned by the test set as nuisance variation rather than isolated as controlled factors. Taken together, these observations suggest that viewpoint mismatch is a more important design consideration than colour representation under the conditions evaluated in this study.

### 5.2. Model Scaling and Efficient Model Selection: Can a Larger Model Solve That Problem?

The comparable mAP_50_ obtained across all five YOLOv8 variants (Section 4.2) indicates that increasing model capacity provides little additional benefit for the evaluated 18-class frontal-view gesture detection task. This observation is consistent with previous HGR studies reporting competitive performance with lightweight architectures [3,15]. In our experiments, a 21× reduction in parameter count produced no measurable difference in mAP_50_, supporting the selection of YOLOv8n for resource-constrained deployment.

This result should be interpreted as task-specific rather than as evidence that model capacity is generally unimportant for HGR. Differences among the YOLOv8 variants remained visible in mAP_50–95_, indicating that larger models provide more precise bounding-box localisation. For the present application, however, the downstream objective is reliable gesture recognition rather than precise hand localisation; therefore, mAP_50_ is the more operationally relevant metric. The observed behaviour should not be extrapolated to substantially larger gesture vocabularies, fine-grained sign-language recognition, or applications requiring highly accurate localisation.

### 5.3. Edge Deployment and Privacy: Can the Resulting Lightweight Solution Actually Be Deployed?

The deployment experiments demonstrate that the proposed HGR pipeline can operate entirely on resource-constrained edge hardware using platform-specific inference backends, and the controlled measurements of Section 4.6 characterise how well it does so. The same lightweight detector was deployed using NCNN on Raspberry Pi 5 and TensorRT on Jetson Orin Nano, illustrating the portability of the pipeline across CPU- and GPU-oriented embedded platforms. The exported inference engines preserved the detection performance of the original model at the validation level, indicating that conversion to the hardware-specific deployment format did not introduce measurable accuracy degradation in the evaluated configuration.

The observed processing rate on Raspberry Pi 5 was sufficient for the temporal verification mechanism used in the proposed smart-home pipeline. Importantly, this application does not require inference at the native camera frame rate: control commands are generated only after a gesture remains stable across the processed frames within the verification interval. This provides a practical mechanism for combining lightweight inference with robust command generation on low-power hardware, and it is also what makes the thermal degradation reported in Section 4.6 tolerable: because the verification gates are expressed as fractions of the frames actually processed rather than as fixed frame counts, the decision rule remains applicable as throughput changes. Reduced throughput may nevertheless increase realised activation latency, an effect that was not quantified directly in the present study.

The thermal results nonetheless carry a concrete deployment implication. A passively cooled Raspberry Pi 5 running this workload continuously reaches its throttling threshold within the first minutes and had not stabilised after ten minutes, so sustained residential operation should assume either active cooling, a duty-cycled inference schedule, or the lower sustained throughput indicated by the second-half plateau rather than the figures obtained from short benchmark runs. The Jetson Orin Nano exhibited no such behaviour under the same workload. We note that this contrast concerns thermal headroom rather than energy cost, which we did not measure.

Fully on-device processing also addresses an important privacy requirement of camera-based smart-home systems. Hand images may contain biometric information when processed for identification purposes, and continuous transmission of residential video to external servers may therefore raise privacy and user-acceptance concerns. In the proposed pipeline, frames are processed locally and discarded immediately after inference; no video data need to be stored or transmitted to an external server. The edge implementation therefore contributes not only to computational feasibility but also to privacy-preserving operation in residential environments.

### 5.4. Limitations of the Study

Several limitations should be considered when interpreting the findings.

First, although CeilGest comprises 156,282 annotated frames, the data were collected from 68 participants, substantially fewer than the 37,583 contributors represented in HaGRID. Subject-level train/validation/test splitting prevents identity leakage and, because each participant was recorded in a separate home, also guarantees that training and test environments are disjoint; nevertheless, the participant pool remains small relative to large-scale public benchmarks, which may restrict generalisation to broader populations. A further consequence of the one-participant-per-home acquisition design is that subject identity and recording environment are perfectly confounded: the held-out results cannot separate generalisation to unseen individuals from generalisation to unseen rooms. Disentangling the two would require recording several participants per environment, or the same participant across several environments.

Second, consecutive frames within each gesture repetition are temporally correlated. Although participants lowered and re-positioned their hands between repetitions, the effective number of independent observations is substantially smaller than the raw frame count. Near-perfect per-frame performance of YOLOv8n_ceil_ should therefore be interpreted as strong detection consistency on the held-out recordings rather than as accuracy over 27,000 independent observations. The principal domain-shift finding relies instead on paired comparisons of models on identical footage, including the approximately 24× difference in class-to-class misclassifications.

Third, each model configuration was trained once; repeated-seed experiments and confidence intervals were not available. Small differences between models should therefore not be interpreted as statistically significant. Viewpoint, model capacity, architecture, and colour space were examined in separate analyses rather than through a fully factorial design. Subject-to-camera distance and illumination, by contrast, varied naturally across the 68 recording environments and were not manipulated as controlled experimental factors. The synthesis of these observations should consequently be interpreted as engineering guidance under the evaluated conditions rather than as a controlled quantitative ranking of independent effect sizes.

Fourth, the objective of this study was to quantify the frontal-to-ceiling domain gap and establish an in-domain supervised baseline using CeilGest. Domain adaptation approaches, including domain-adversarial training and pseudo-label self-training, were not evaluated. The improvement obtained with CeilGest should therefore be interpreted relative to frontal-only training with no adaptation and does not establish that supervised in-domain training is superior to domain adaptation.

Fifth, the temporal-verification parameters were not characterised by a full factorial sweep. Section 3.2 reports the empirical basis for τ and provides a frame-budget interpretation of α and β. The 2 s verification window defines the nominal observation interval, whereas τ, α, and β may influence realised activation latency by determining whether a given window satisfies the confirmation criteria. We do not report measured false-activation rates and end-to-end activation latencies across a grid of (τ,α,β). Such an analysis requires time-ordered per-frame detector outputs, which were not available for this revision, and remains an important extension of the present work.

Sixth, the gesture vocabulary is restricted to 18 static postures. Dynamic and sequential gestures, which would require temporal modelling beyond the sliding-window verification used here, were not addressed, and the conclusions of this study should not be extrapolated to them or to larger vocabularies such as fine-grained sign language.

Seventh, the acquisition conditions themselves are narrow. All recordings used a ceiling mount between 2.5 and 3.0 m with a single camera type and a near-vertical downward view; other mounting heights, oblique ceiling angles, wide-angle or infrared optics, and low-light or night-time operation were not evaluated. The reported robustness therefore applies to the geometry and illumination range spanned by the 68 recording environments and should not be extrapolated beyond it.

Finally, the edge characterisation remains incomplete. Inference latency, sustained throughput, and thermal behaviour were measured under controlled conditions (Section 4.6), but power consumption and energy per inference were not measured. Reduced-precision inference was evaluated using FP16 on the Jetson Orin Nano, whereas INT8 quantisation was not evaluated. The two platforms therefore cannot be compared directly in terms of energy efficiency. Both devices were passively cooled during the reported experiments; the degradation observed on the Raspberry Pi 5 further indicates that sustained deployment on that platform may require active cooling or duty-cycled operation.

### 5.5. Future Work

Future work should address three main directions. First, the supervised in-domain baseline established in this study should be compared with domain adaptation strategies, including adversarial domain alignment and pseudo-label self-training, to determine how much of the frontal-to-ceiling gap can be recovered without labelled target-domain data. Second, CeilGest should be expanded to include a larger and more diverse participant population, additional domestic environments, and naturalistic non-gesture activity. Evaluation at the level of distinct gesture instances and subject-level cross-validation would provide stronger estimates of generalisation and enable analysis of false activation rates during everyday activity. Finally, the edge implementation should be characterised through controlled measurements of latency, sustained throughput, power consumption, and thermal behaviour including direct evaluation of any associated accuracy changes.

## 6. Conclusions

This study presented a privacy-preserving, ceiling-mounted hand gesture recognition system for smart-home control and provided what is, to the best of our knowledge, the first systematic quantification of the frontal-to-ceiling domain gap for YOLO-based HGR across an 18-class static gesture vocabulary. To support this investigation, we collected and manually annotated CeilGest, a ceiling-view dataset comprising 156,282 annotated frames from 68 participants.

The principal finding is that viewpoint mismatch can substantially degrade gesture recognition even when aggregate detection metrics remain high. On identical ceiling-view footage, the frontal-trained YOLOv8n produced approximately 24× more class-to-class misclassifications than its in-domain counterpart. This is a paired frame-level comparison on identical footage, and the substantially greater error burden of the frontal-trained model remains evident when temporal dependence within gesture holds is taken into account. However, the hold-level quantities reported in Section 4.3 are analytical estimates derived from frame-level counts rather than an independent empirical re-estimation of the exact error ratio. Accordingly, absolute aggregate detection metrics should not be interpreted as estimates based on 27,000 statistically independent observations. The effect was strongly class-dependent, with AP decreasing by up to 5.5 percentage points for the worst-affected gesture (call). The observed confusions were geometrically interpretable, particularly for gestures distinguished by thumb orientation and finger configuration, highlighting the importance of class-wise analysis when evaluating HGR under viewpoint changes.

We emphasise that this establishes an in-domain supervised baseline rather than an optimum: mainstream domain-adaptation methods were not evaluated against the frontal-to-ceiling gap, so the improvement reported here is measured relative to no adaptation at all, and a comparison against those methods remains the principal open question (Section 5.5).

Increasing model capacity provided limited benefit under the evaluated conditions. Five YOLOv8 variants achieved similar mAP_50_ on HaGRID, supporting selection of the lightweight YOLOv8n, while viewpoint-matched training produced substantially fewer ceiling-view errors than increasing the capacity of frontal-trained models. Among the factors examined, these findings identify training–deployment viewpoint consistency as a key consideration for HGR systems operating under non-standard camera geometries.

Finally, the complete pipeline was deployed and characterised on Raspberry Pi 5 using NCNN and Jetson Orin Nano using TensorRT. Processing was performed entirely on-device, allowing acquired frames to be discarded after inference without transmission to an external server. Controlled measurement showed that the two platforms differ sharply under sustained load: a passively cooled Raspberry Pi 5 degraded by 17.6% within ten minutes and had not stabilised, so continuous residential operation on that platform would require active cooling or duty-cycled inference. Power consumption and INT8 inference were not evaluated. Together, the results demonstrate the feasibility of lightweight, viewpoint-aware, and privacy-preserving HGR for ceiling-mounted smart-home applications, subject to the limitations discussed in Section 5.4.

## Figures and Tables

**Table 1:** Comparison of representative HGR methods. “Metric” identifies each reported figure to avoid misleading cross-metric comparisons. Bold indicates the present study.

Authors	Model	Dataset	Scale	Cls.	Best	Metric	View	Platform	Limitation
Gao et al. [9]	v9t/v10n	TSL Det.	5 k	31	99.0	mAP_50_	Front.	Desktop GPU	Single dataset
Alabdullah et al. [4]	Feat.+RNN	HaGRID	553 k	7	92.6	Cls. acc.	Front.	Desktop GPU	No edge
Ahuja et al. [25]	MobileNet	Custom	20 k	10	93.0	Cls. acc.	Front.	Desktop GPU	Controlled bg.
Hobbs & Ali [18]	MP+FFN	HaGRIDv2	1.636 M	15	90.0	Cls. acc.	Front.	RPi 5 (CPU)	Skeleton; CPU
Lopes et al. [19]	MediaPipe	MP pretr.	16 k	7	95.5	$F_{1}$	Front.	Android	No HW accel.
Beşenk et al. [5]	v8s+MNv3	HaGRID	554 k	18	99.1	Cls. acc.	Front.	Desktop GPU	No edge
**This work**	v8n/v10n	HaGRID+Ceil.	710 k	18	>99	mAP_50_	Ceil.	Desktop GPU, RPi 5 + Jetson	static gestures

**Table 2:** Classification model comparison on HaGRID (90,000 images, 18 classes). The RGB, YCbCr and HSV columns report *top-1 classification accuracy* on a balanced held-out set, evaluated at 224 × 224 without localisation. These values are not comparable with the detection metrics reported in Section 4, which are mean average precision computed over predicted bounding boxes; the two quantities answer different questions and should not be read as a single ranking.

Model	Size (MB)	Params (M)	RGB	YCbCr	HSV
MobileNetV3Small	6.6	2.5	0.9855	0.9812	0.9804
MobileNetV3Large	16.4	5.5	0.9905	0.9883	0.9861
EfficientNetV2	89.0	21.5	0.9867	0.9844	0.9805
ResNet18	43.8	11.7	0.9800	0.9765	0.9734
YOLOv8n	6.5	3.16	**0.9960**	0.9910	0.9895

**Table 3:** YOLOv8 variant comparison on HaGRID (frontal perspective). Cells marked “—” indicate configurations not evaluated due to GPU memory constraints. All measurements on an NVIDIA RTX 4090 desktop GPU (NVIDIA Corporation, Santa Clara, CA, USA) with batch inference. Precision (P) and recall (R) are reported at IoU = 0.50 and are detection metrics, not classification accuracy.

					mAP_50_			mAP_50–95_		
Model	Params	GFLOPs	P	R	bs 16	bs 32	bs 64	bs 16	bs 32	bs 64
YOLOv8n	3.16 M	8.9	0.996	0.995	0.995	0.995	0.995	0.897	0.897	0.897
YOLOv8s	11.17 M	28.8	0.997	0.997	0.995	0.995	0.995	0.903	0.905	0.903
YOLOv8m	25.90 M	79.3	0.997	0.998	0.995	0.995	—	0.906	0.906	—
YOLOv8l	43.69 M	165.7	0.998	0.998	0.995	—	—	0.907	—	—
YOLOv8x	68.23 M	258.5	0.998	0.998	0.995	—	—	0.908	—	—

**Table 4:** Hold-level sensitivity analysis derived from the frame-level misclassification counts reported in Section 4.4. Holds correspond to ≈5 s gesture repetitions (approximately 900 holds in total and approximately 30 sampled frames per hold). “Max. holds” gives the maximum number of majority-misclassified holds possible from the observed frame-level errors, assuming that each affected hold contains the minimum number of errors required for majority misclassification. “Est. holds” provides an approximate value under the assumption that errors are concentrated within holds. These quantities are analytical estimates derived from frame-level counts and do not constitute an independent empirical hold-level evaluation.

Model	Frame Errors	Max. Holds	Est. Holds	95% CI, Hold Error Rate
YOLOv8n_ceil_	20	1	0.7	[0.00%, 0.62%]
YOLOv8n_HaGRID_	486	30	16.2	[1.02%, 2.87%]
YOLOv10n_HaGRID_	761	47	25.4	[1.81%, 4.07%]
YOLOv10x_HaGRID_	206	12	6.9	[0.31%, 1.60%]

**Table 5:** Per-frame detection consistency on the ceiling test set (18 classes, excl. no_gesture). All columns are detection metrics evaluated per frame: mAP_50_ and mAP_50–95_ are mean average precision at IoU = 0.50 and averaged over IoU = 0.50–0.95 respectively, while P, R and F1 are computed at IoU = 0.50. As set out in Section 4.3, absolute values and small differences in the second or third decimal place should not be read as a performance ranking.

Model	mAP_50_	mAP_50–95_	P	R	F1
YOLOv8n_ceil_	0.997	0.965	0.979	1.000	0.989
YOLOv8n_HaGRID_	0.991	0.960	0.960	0.989	0.974
YOLOv10n_HaGRID_	0.989	0.923	0.944	0.961	0.952
YOLOv10x_HaGRID_	0.997	0.931	0.981	0.984	0.982

**Table 6:** Domain-gap performance on the 18-class CeilGest ceiling-view test set (27,000 images). All models are evaluated on the same test footage. “Dominant Confusion” reports the most frequent class-to-class error for each model, while “Total Mis.” counts all class-to-class off-diagonal entries in the corresponding confusion matrix (Figure 3); the background/missed-detection column is excluded.

Model	Training View	mAP_50_	Worst Class (AP)	Dominant Confusion	Total Mis.
YOLOv8n_ceil_	Ceiling	0.997	like (0.999)	ok→four (5)	20
YOLOv8n_HaGRID_	Frontal	0.991	call (0.945)	call→like (139)	486
YOLOv10n_HaGRID_	Frontal	0.989	call (0.979)	call→like (151)	761
YOLOv10x_HaGRID_	Frontal	0.997	one (0.985)	mute→like (39)	206

**Table 7:** Summary of the factors investigated for ceiling-view HGR, their observed effects, and practical implications. The factors were evaluated in separate experiments rather than through a fully factorial design; therefore, the observations should be interpreted as qualitative engineering guidance rather than a quantitative ranking of effect sizes.

Factor	Comparison	Main Observation	Practical Implication
Training-domain match	Frontal-trained vs. ceiling-trained	On identical ceiling footage, frontal-trained YOLOv8n produced ≈24× more class-to-class misclassifications than ceiling-trained YOLOv8n (486 vs. 20); the worst-affected class (call) showed a 5.5-percentage-point AP decrease.	Matching the training viewpoint to the deployment viewpoint substantially reduces ceiling-view recognition errors.
Model capacity	YOLOv8n → YOLOv8x	All five YOLOv8 variants achieved similar frontal-view performance (mAP_50_ ≈ 0.995), with less than 1.1% variation in mAP_50–95_.	Increasing model capacity provides limited benefit when detection performance is already saturated, supporting the use of lightweight models.
Architecture	YOLOv8 vs. YOLOv10	Different error patterns and robustness were observed when frontal-trained detectors were evaluated on ceiling-view data.	Detector architecture can influence robustness to viewpoint-induced domain shift.
Colour space	RGB vs. HSV vs. YCbCr	Differences between colour representations were below 0.7 percentage points in accuracy in the classification experiments.	Colour representation had a limited effect under the evaluated conditions.

**Table 8:** Consolidated findings summary across all experimental subsections.

Finding	Evidence	Caveat/Scope
Metric ceiling at mAP_50_	All five YOLOv8 variants achieve 0.995 on HaGRID (Section 4.2)	Specific to 18-class frontal gesture task at IoU = 0.50; gap emerges at mAP_50–95_
YOLOv8n suffices for edge deployment	21× smaller, 29× fewer FLOPs than YOLOv8x with identical mAP_50_	Holds for frontal HaGRID; ceiling data required separate training
Domain shift is the most robust effect among factors examined	≈24× more frame-level misclassifications on identical footage; large paired difference remains evident after accounting for within-hold temporal dependence; up to 5.5 pp AP drop on the worst class (Section 4.4)	Non-factorial design; ranking is indicative, not controlled; mean mAP_50_ effect milder (0.6 pp for the same-architecture paired comparison, Table 5), comparable to capacity
In-domain data closes the gap	YOLOv8n_ceil_ reduces misclassifications by more than an order of magnitude (≈486→20 errors/27,000 frames)	≈50 separate gesture holds per class, clustered within 10 held-out subjects (10 subjects × ≈5 reps); absolute near-ceiling mAP not interpreted; trained from COCO-pretrained weights, not fine-tuned from HaGRID; DA methods not compared
Physically interpretable confusions	call↔like (thumb occlusion), finger-count gestures (foreshortening)	Specific to near-vertical overhead angle
Larger models partially compensate	YOLOv10x reduces call→like from 139 to 6 errors (total 206 vs. 486)	At 11× the parameters and ≈20× the FLOPs; still inferior to in-domain nano
On-device operation validated and characterised	Inference 74.69 ms (RPi 5, NCNN) and 12.72 ms (Jetson, TensorRT); RPi 5 degrades 17.6% over ten minutes, reaching 90.8 °C	Power consumption and INT8 inference not evaluated; RPi 5 figures obtained at 640 × 480 capture (Section 4.6)
Colour space negligible	<0.7 pp variation across RGB/HSV/YCbCr	Single-run comparison on 224×224 classification only

## Data Availability

The data presented in this study are available on reasonable request from the corresponding author and after signing a waiver form due to privacy restrictions.

## Abbreviations

The following abbreviations are used in this manuscript:

HGR	Hand Gesture Recognition
DA	Domain Adaptation
mAP	mean Average Precision
AP	Average Precision
CI	Confidence Interval
FPS	Frames Per Second
GDPR	General Data Protection Regulation
UART	Universal Asynchronous Receiver–Transmitter
CSI	Camera Serial Interface
NMS	Non-Maximum Suppression
INT8	8-bit Integer Quantisation
FP16	16-bit Floating Point
RPi	Raspberry Pi
TDP	Thermal Design Power
